# High density IgE recognition of the major grass pollen allergen, Phl p 1, revealed with single chain IgE antibody fragments obtained by combinatorial cloning

**DOI:** 10.1186/2045-7022-4-S2-P18

**Published:** 2014-03-17

**Authors:** Christoph Madritsch, Elisabeth Gadermaier, Christian Lupinek, Rudolf Valenta, Flicker Sabine

**Affiliations:** 1 Medical University of Vienna, Center for Pathophysiology, Infectiology & Immunology, Vienna, Austria

## Background

Grass pollen is one of the most potent and frequently recognized allergen sources. The timothy grass pollen allergen Phl p 1 belongs to the group 1 of highly cross-reactive grass pollen allergens with a molecular weight of approximately 25-30 kDa. Group 1 allergens are recognized by more than 95% of grass pollen allergic patients. A major mechanism of allergic inflammation is the cross-linking of mast cell and basophil-bound IgE antibodies by allergens.

## Methodes

Here we investigated the IgE recognition of Phl p 1 using allergen specific single chain IgE antibody fragments (IgE-ScFvs) obtained by combinatorial cloning from a combinatorial IgE-ScFv library constructed from PBMC of a grass pollen allergic patient.

## Results

Using phage display, two Phl p 1-specific ScFv with high specificity and affinity for Phl p 1 were isolated and their binding sites were found to be localized with synthetic Phl p 1-derived peptides in close vicinity at the N-terminus of the allergen. Moreover, we could show by surface plasmon resonance experiments that both IgE-ScFvs could simultaneously bind to Phl p 1 without notable steric hindrance. Even when we used a combination of the two IgE-ScFvs and an additional human Phl p 1-specific IgE, no relevant inhition of allergic patients polyclonal IgE binding to Phl p 1 could be achieved, indicating high density IgE recognition of the Phl p 1 allergen by multiple IgE antibodies.

## Conclusion

Our results indicate that allergic patients IgE antibodies can bind in an unusual density without steric hindrance to Phl p 1 which may explain the high allergenic activity of this allergen.

